# ARMOR: An Automated Reproducible MOdular Workflow for Preprocessing and Differential Analysis of RNA-seq Data

**DOI:** 10.1534/g3.119.400185

**Published:** 2019-05-14

**Authors:** Stephany Orjuela, Ruizhu Huang, Katharina M. Hembach, Mark D. Robinson, Charlotte Soneson

**Affiliations:** * SIB Swiss Institute of Bioinformatics, Zurich, Switzerland; † Institute of Molecular Life Sciences, University of Zurich, Zurich, Switzerland; ‡ Institute of Molecular Cancer Research, University of Zurich, Zurich, Switzerland; § Department of Quantitative Biomedicine, University of Zurich, Zurich, Switzerland

**Keywords:** RNA sequencing, Differential expression, Exploratory data analysis, Quality control

## Abstract

The extensive generation of RNA sequencing (RNA-seq) data in the last decade has resulted in a myriad of specialized software for its analysis. Each software module typically targets a specific step within the analysis pipeline, making it necessary to join several of them to get a single cohesive workflow. Multiple software programs automating this procedure have been proposed, but often lack modularity, transparency or flexibility. We present ARMOR, which performs an end-to-end RNA-seq data analysis, from raw read files, via quality checks, alignment and quantification, to differential expression testing, geneset analysis and browser-based exploration of the data. ARMOR is implemented using the Snakemake workflow management system and leverages conda environments; Bioconductor objects are generated to facilitate downstream analysis, ensuring seamless integration with many R packages. The workflow is easily implemented by cloning the GitHub repository, replacing the supplied input and reference files and editing a configuration file. Although we have selected the tools currently included in ARMOR, the setup is modular and alternative tools can be easily integrated.

Since the first high-throughput RNA-seq experiments about a decade ago, there has been a tremendous development in the understanding of the characteristic features of the collected data, as well as the methods used for the analysis. Today there are vetted, well-established algorithms and tools available for many aspects of RNA-seq data analysis (Conesa *et al.* 2016; Van Den Berge *et al.* 2018). In this study, we capitalize on this knowledge and present a modular, light-weight RNA-seq workflow covering the most common parts of a typical end-to-end RNA-seq data analysis with focus on differential expression. The application is implemented using the Snakemake workflow management system (Köster and Rahmann 2012), and allows the user to easily perform quality assessment, adapter trimming, genome alignment, transcript and gene abundance quantification, differential expression analysis and geneset analyses with a simple command, after specifying the required reference files and information about the experimental design in a configuration file. Reproducibility is ensured via the use of conda environments, and all relevant log files are retained for transparency. The output is provided in state-of-the-art R/Bioconductor objects, ensuring interoperability with a broad range of Bioconductor packages. In particular, we provide a template to facilitate browser-based interactive visualization of the quantified abundances and the results of the statistical analyses with iSEE (Rue-Albrecht *et al.* 2018).

Among already existing pipelines for automated reference-based RNA-seq analysis, several focus either on the preprocessing and quality control steps (He *et al.* 2018; Ewels *et al.* 2018; Tsyganov *et al.* 2018), or on the downstream analysis and visualization of differentially expressed genes (Marini 2018; Monier *et al.* 2019; Powell 2018), or do not provide a single framework for the preprocessing and downstream analysis (Steinbaugh *et al.* 2018). Some workflows are based on predefined reference files and can only quantify abundances for human or mouse (Torre *et al.* 2018; Cornwell *et al.* 2018; Wang 2018). Additionally, workflows that conduct differential gene expression analysis often do not allow comparisons between more than two groups, or more complex experimental designs (Girke 2018; Cornwell *et al.* 2018). Some existing pipelines only provide a graphical user interface to design and execute fully automated analyses (Hung *et al.* 2018; Afgan *et al.* 2018). In addition to reference-based tools, there are also pipelines that perform *de novo* transcriptome assembly before downstream analysis (*e.g.*, https://github.com/dib-lab/elvers).

ARMOR performs both preprocessing and downstream statistical analysis of the RNA-seq data, building on standard statistical analysis methods and commonly used data containers. It distinguishes itself from existing workflows in several ways: (i) Its modularity, reflected in its fully and easily customizable framework. (ii) The transparency of the output and analysis, meaning that all code is accessible and can be modified by the user. (iii) The seamless integration with downstream analysis and visualization packages, especially those within Bioconductor (Huber *et al.* 2015; Amezquita *et al.* 2019). (iv) The ability to specify any fixed-effect experimental design and any number of contrasts, in a standardized format. (v) The inclusion of a test for differential transcript usage in addition to differential gene expression analysis. While high-performance computing environments and cloud computing are not specifically targeted, Snakemake enables the usage of a cluster without the need to modify the workflow itself.

In general, we do not advocate fully automated analysis. All rigorous data analyses need exploratory steps and spot checks at various steps throughout the process, to ensure that data are of sufficient quality and to spot potential errors (*e.g.*, sample mislabelings). ARMOR handles the automation of ”bookkeeping” tasks, such as running the correct sequence of software for all samples, and compiling the data and reports in standardized formats. If errors are identified, the workflow can re-run only the parts that need to be updated.

ARMOR is available from https://github.com/csoneson/ARMOR.

## Materials and Methods

### Overview

**Figure 1:**
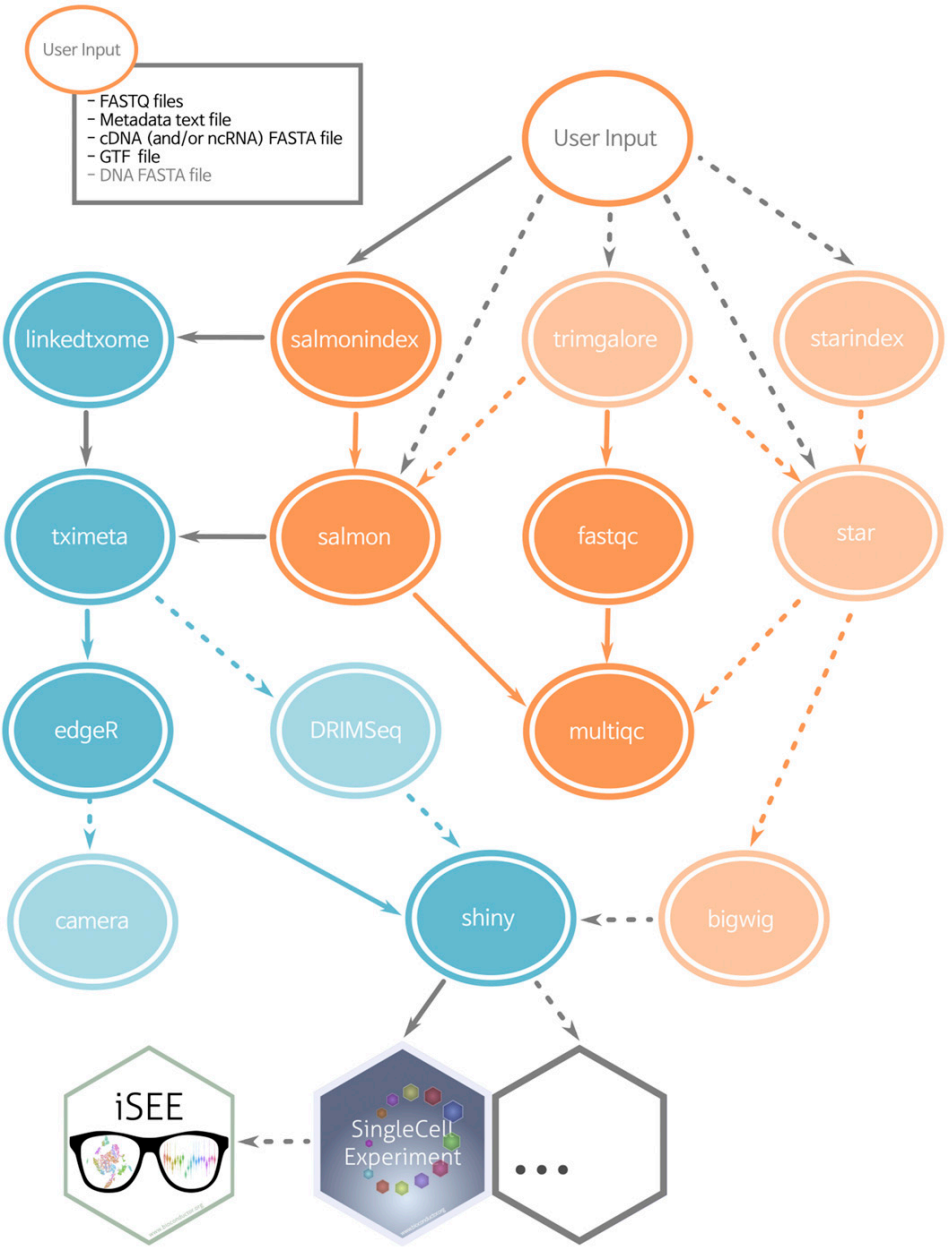
Simplified directed acyclic graph (DAG) of the ARMOR workflow. Blue ellipses are rules run in R, orange ellipses from software called as shell commands. Dashed lines and light-colored ellipses are optional rules, controlled in the configuration file. By default all rules are executed.

The ARMOR workflow is designed to perform an end-to-end analysis of bulk RNA-seq data, starting from FASTQ files with raw sequencing reads (Figure 1). Reads first undergo quality control with FastQC (https://www.bioinformatics.babraham.ac.uk/projects/fastqc/) and (optionally) adapter trimming using TrimGalore! (https://www.bioinformatics.babraham.ac.uk/projects/trim_galore/), before being mapped to a transcriptome index using Salmon (Patro *et al.* 2017) and (optionally) aligned to the genome using STAR (Dobin *et al.* 2013). Estimated transcript abundances from Salmon are imported into R using the tximeta package (Soneson *et al.* 2015; Love *et al.* 2019) and analyzed for differential gene expression and (optionally) differential transcript usage with edgeR (Robinson *et al.* 2010) and DRIMSeq (Nowicka and Robinson 2016). The quantifications, provided metadata, and results from the statistical analyses are exported as SingleCellExperiment objects (Lun and Risso 2019) ensuring interoperability with a large part of the Bioconductor ecosystem (Huber *et al.* 2015; Amezquita *et al.* 2019). Quantification and quality control results are summarized in a MultiQC report (Ewels *et al.* 2016). Other tools can be easily exchanged for those listed above by modifying the Snakefile and/or the template analysis code.

### Input file specification

ARMOR can be used to analyze RNA-seq data from any organism for which a reference transcriptome and (optionally) an annotated reference genome are available from either Ensembl (Zerbino *et al.* 2018) or GENCODE (Frankish *et al.* 2019). Paths to the reference files, as well as the FASTQ files with the sequencing reads, are specified by the user in a configuration file. In addition, the user prepares a metadata file – a tab-delimited text file listing the name of the samples, the library type (single- or paired-end) and any other covariates that will be used for the statistical analysis. The checkinputs rule in the Snakefile can be executed to make sure all the input files and the parameters in the configuration file have been correctly specified.

### Workflow execution

ARMOR is implemented as a modular Snakemake (Köster and Rahmann 2012) workflow, and the execution of the individual steps is controlled by the provided Snakefile. Snakemake will automatically keep track of the dependencies between the different parts of the workflow; rerunning the workflow will thus only regenerate results that are out of date or missing given these dependencies. Via a set of variables specified in the configuration file, the user can easily decide to include or exclude the optional parts of the workflow (shaded ellipses in Figure 1). By adding or modifying targets in the Snakefile, users can include any additional or specialized types of analyses that are not covered by the original workflow.

By default, all software packages that are needed for the analysis will be installed in an auto-generated conda environment, which will be automatically activated by Snakemake before the execution of each rule. The desired software versions can be specified in the provided environment file. If the user prefers, local installations of (all or a subset of) the required software can also be used (as described in **Software management**).

### Software management

First, the user needs to have a recent version of Snakemake and conda installed. There is a range of possibilities to manage the software for the ARMOR workflow. The recommended option is to allow conda and the workflow itself to manage everything, including the installation of the needed R packages. The workflow is executed this way with the command

snakemake‐‐use-conda

The first time the workflow is run, the conda environments will be generated and all necessary software will be installed. Any subsequent invocations of the workflow from this directory will use these generated environments. An alternative option is to use ARMOR’s envs/environment.yaml file to create a conda environment that can be manually activated, by running the command

conda env create‐‐name ARMOR \

‐‐file envs/environment.yaml

conda activate ARMOR

The second command activates the environment. Once the environment is activated, ARMOR can be run by simply typing

snakemake

Additionally, the user can circumvent the use of conda, and make sure that all software is already available and in the user’s PATH. For this, Snakemake and the tools listed in envs/environment.yaml need to be manually installed, in addition to a recent version of R and the R packages listed in scripts/install_pkgs.R.

For either of the options mentioned above, the useCondaR flag in the configuration file controls whether a local R installation, or a conda-installed R, will be used. If useCondaR is set to False, the path to a local R installation (*e.g.*, Rbin:<path>) must be specified in the configuration file, along with the path to the R package library (*e.g.*, R_LIBS_USER=“<path>”) in the .Renviron file. If the specified R library does not contain the required packages, Snakemake will try to install them (*i.e.*, write permissions would be needed). ARMOR has been tested on macOS and Linux systems.

### Statistical analysis

ARMOR uses the quasi-likelihood framework of edgeR (Robinson *et al.* 2010; Lun *et al.* 2016) to perform tests for differential gene expression, camera (Wu and Smyth 2012) to perform associated geneset analysis, and DRIMSeq (Nowicka and Robinson 2016) to test for differential transcript usage between conditions. All code to perform the statistical analyses is provided in Rmarkdown templates (Allaire *et al.* 2018; Xie *et al.* 2018), which are executed at runtime. This setup gives the user flexibility to use any experimental design supported by these tools, and to test any contrast(s) of interest, by specifying these in the configuration file using standard R syntax, *e.g.*,

design:“^∼^ 0 + group”

contrast:groupA-groupB

Arbitrarily complex designs and multiple contrasts are supported. In addition, by editing the template code, users can easily configure the analysis, add additional plots, or even replace the statistical test if desired. After compilation, all code used for the statistical analysis, together with the results and version information for all packages used, is retained in a standalone html report, ensuring transparency and reproducibility and facilitating communication of the results.

### Output files

The output files from all steps in the ARMOR workflow are stored in a user-specified output directory, together with log files for each step, including relevant software version information. A detailed summary of the output files generated by the workflow, including the shell command that was used to generate each of them, the time of creation, and information about whether the associated inputs, code or parameters have since been updated, can be obtained at any time by invoking Snakemake with the flag -D (or‐‐detailed-summary). Using the benchmark directive of Snakemake, ARMOR also automatically generates additional text files summarizing the run time and peak memory usage of each executed rule.

The results from the statistical analyses are combined with the transcript- and gene-level quantifications and saved as SingleCellExperiment objects (Lun and Risso 2019), ensuring easy integration with a large number of Bioconductor packages for downstream analysis and visualization. For example, the results can be interactively explored using the iSEE package (Rue-Albrecht *et al.* 2018) and a template is provided for this.

### Multiple project management

When managing multiple projects, the user might run ARMOR in multiple physical locations (*i.e.*, clone the repository in separate places). snakemake‐‐use-conda will create a separate conda environment in each subdirectory, which means that the installed software may be duplicated. If disk space is a concern, building and activating a single conda environment (using the conda env create command as shown in the **Software management** section), and activating this before invoking each workflow may be beneficial. It is also possible to explicitly specify the path to the desired config.yaml configuration file when snakemake is called:

snakemake‐‐configfile config.yaml

Thus, the same ARMOR installation can be used for multiple projects, by invoking it with a separate config.yaml file for each project.

**Figure 2:**
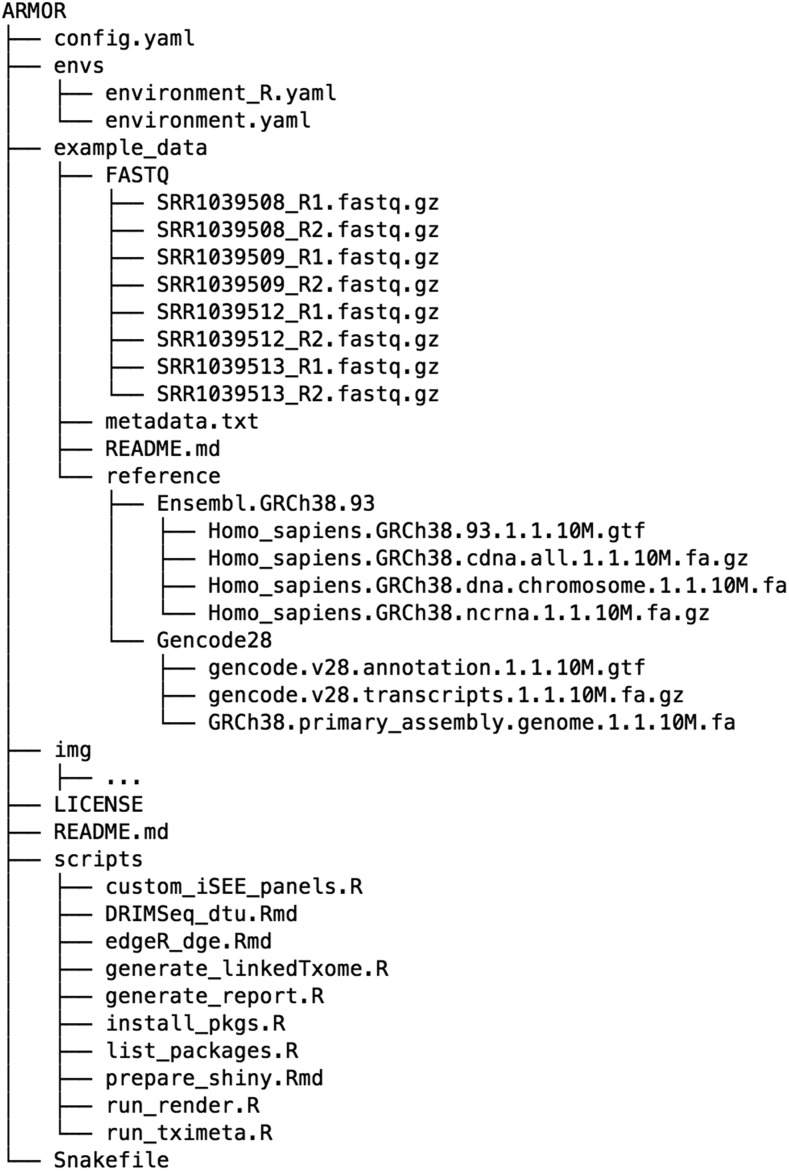
The files and directory structure that make up the ARMOR workflow.

By taking advantage of the Snakemake framework, ARMOR makes file and software organization relatively autonomous. Although we recommend using a file structure similar to the one used for the example data provided in the repository (Figure 2), and managing all the software for a project in a conda environment, the user is free to use the same environment for different datasets, even if the files are located in several folders. This alternative is more of a “software-based” structure than the “project-based” structure we present with the pipeline. Either structure has its advantages and depending on the use case and level of expertise, both can be easily implemented using ARMOR.

### Code availability

ARMOR is available (under MIT license) from https://github.com/csoneson/ARMOR, together with a detailed walk-through of an example analysis. The repository also contains a wiki (https://github.com/csoneson/ARMOR/wiki), which is the main source of documentation for ARMOR and contains extensive information about the usage of the workflow.

### Data Availability

Supplemental file DataS1.html contains the MultiQC report for the data used in the **Real data walk-through** section (ArrayExpress accession number E-MTAB-7029). Supplemental material available at FigShare: https://doi.org/10.25387/g3.8053280.

## Results and Discussion

### The ARMOR skeleton

Figure 2 shows the set of files contained within the ARMOR workflow, and what is downloaded to the user’s computer when the repository is cloned.

The example_data directory represents a (runnable) template of a very small dataset, which is useful for testing the software setup and the system as well as for having a structure to copy for a real project. The provided config.yaml file is pre-configured for this example dataset. We recommend that users prepare their own config.yaml and a similar directory structure to example_data, with the raw FASTQ files and reference sequence and annotation information in subfolders, perhaps using symbolic links if such files are already available in another location. We present an independent example below in the **Real data walk-through** section.

Once everything is set up, running snakemake, which operates on the rules in the Snakefile, will construct the hierarchy of instructions to execute, given the specifications in the config.yaml file. Snakemake automatically determines the dependencies between the rules and will invoke the instructions in a logical order. The scripts and envs directories, and the Snakefile itself, should not need to be modified, unless the user wants to customize certain aspects of the pipeline.

### Real data walk-Through

Here, we illustrate the practical usage of ARMOR on a bulk RNA-seq dataset from a study on Wnt signaling (Doumpas *et al.* 2019). For each of three genetic backgrounds (HEK 293T, dBcat and d4TCF) and two experimental conditions (untreated and stimulated using the GSK3 inhibitor CHIRON99021), three biological replicates were measured (18 samples in total). The number of sequenced reads for each individual sample ranges from 12.5 to 41 million. A more detailed overview of the dataset is provided in the MultiQC report generated by the ARMOR run (Supplemental File DataS1.html). An R script (download_files.R, which can be found at https://github.com/csoneson/ARMOR/blob/chiron_realdataworkflow/E-MTAB-7029/download_files.R) was written to download the FASTQ files with raw reads from ArrayExpress (https://www.ebi.ac.uk/arrayexpress/experiments/E-MTAB-7029/), and create a metadata table detailing the type of library and experimental condition for each sample (Table 1). This table was saved as a tab-delimited text file named metadata.txt.

**Table 1:** Metadata table for the Wnt signaling data

Names	type	condition
Q10-Chir-1_R1	SE	d4Tcf__chir
Q10-Chir-2_R1	SE	d4Tcf__chir
Q10-Chir-3_R1	SE	d4Tcf__chir
b-cat-KO-Chir-1_R1	SE	dBcat__chir
b-cat-KO-Chir-2_R1	SE	dBcat__chir
b-cat-KO-Chir-3_R1	SE	dBcat__chir
WT-Chir-1_R1	SE	WT__chir
WT-Chir-2_R1	SE	WT__chir
WT-Chir-3_R1	SE	WT__chir
Q10-unstim-1_R1	SE	d4Tcf__unstim
Q10-unstim-2_R1	SE	d4Tcf__unstim
Q10-unstim-3_R1	SE	d4Tcf__unstim
b-cat-KO-ustim-1_R1	SE	dBcat__unstim
b-cat-KO-ustim-2_R1	SE	dBcat__unstim
b-cat-KO-ustim-3_R1	SE	dBcat__unstim
WT-unstim-1_R1	SE	WT__unstim
WT-unstim-2_R1	SE	WT__unstim
WT-unstim-3_R1	SE	WT__unstim

**Figure 3:**
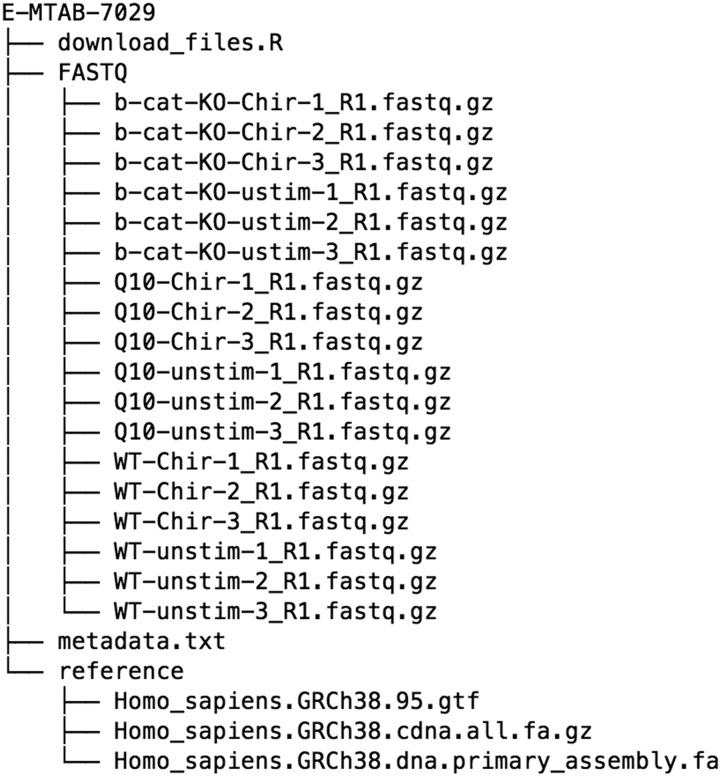
The suggested structure for the set of files that need to be organized to run ARMOR on a new dataset. The structure can deviate from this somewhat, since the location of the files can be specified in the corresponding config.yaml file.

The raw data and reference files were organized into a directory, E-MTAB-7029, with the structure according to Figure 3. The default config.yaml downloaded with the workflow was copied into a new file called config_E-MTAB-7029.yaml and edited to reflect the location of these files. In addition, the read length was set and the experimental design was specified as “∼ 0 + condition”, where the condition information will be taken from metadata.txt. Then, a set of contrasts of interest (*e.g.*, conditiond4Tcf__chir-conditiond4Tcf__unstim) were specified, as well as the set of genesets to use. The final configuration file can be viewed at https://github.com/csoneson/ARMOR/blob/chiron_realdataworkflow/config_E-MTAB-7029.yaml.

The set of files (not including the large data and reference files, which would be downloaded using the download_files.R) used in this setup can be found on the chiron_realdataworkflow branch of the ARMOR repository: https://github.com/csoneson/ARMOR/tree/chiron_realdataworkflow.

After downloading the data, generating the metadata.txt file and editing the config.yaml file, the full workflow was run with the command:

snakemake‐‐use-conda‐‐cores 20 \

**Figure 4:**
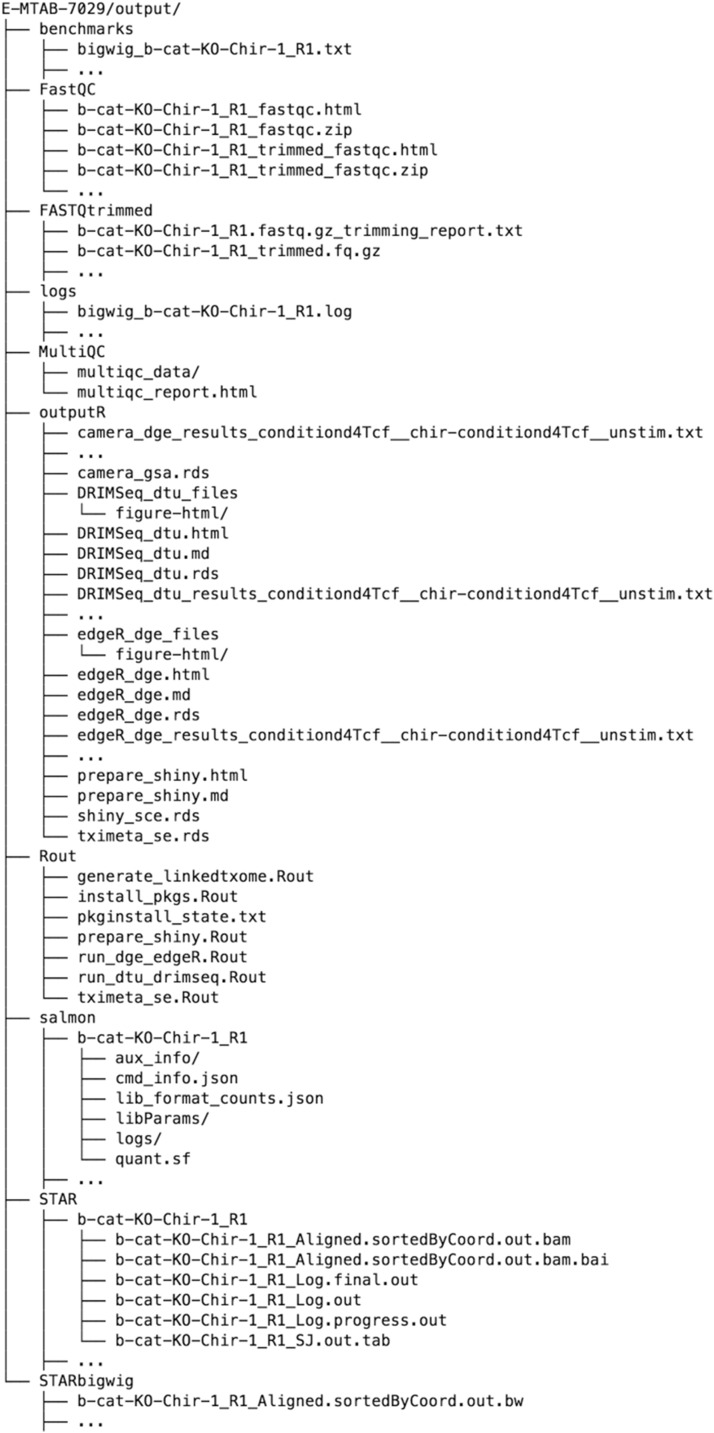
The set of output files from the workflow. This includes log files for every step and all the standard outputs of all the tools, such as R objects and scripts, BAM files, bigWig files and quantification tables. Note that the outputs for only one RNA-seq sample are shown; ... represents the set of output files for the remaining samples or contrasts. Directories ending in / contain extraneous files and are collapsed here.

**Figure 5:**
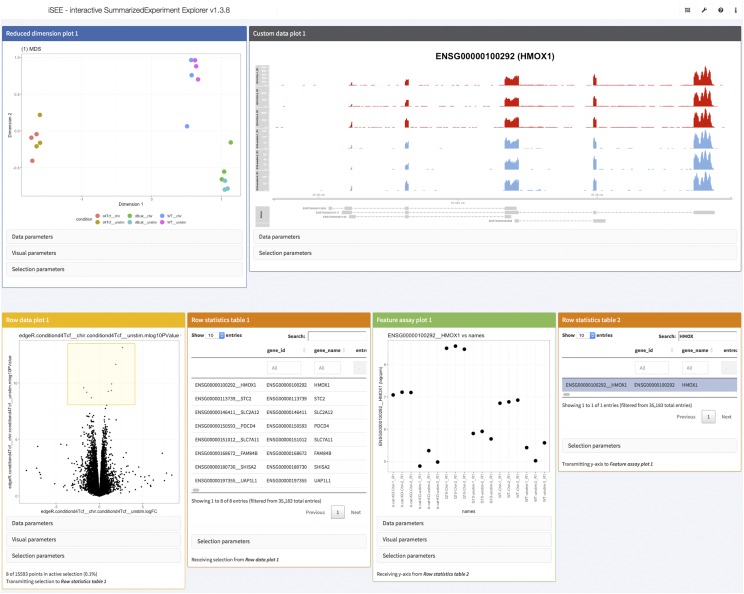
Screenshot of visualization of data and results from the real data walk-through using the iSEE R/Bioconductor package. The interactive application was configured to display an MDS plot colored by the sample condition (top left), a custom panel showing the observed read coverage of a selected gene (top right), a volcano plot for a specified contrast (bottom left, the selected genes are shown in the adjacent table) and an overview of the log-CPM expression values for each sample, for a gene selected in a second table (bottom right).

‐‐configfile config_E-MTAB-7029.yaml

and upon completion of the workflow run, the specified output directory was populated as shown in Figure 4. The MultiQC directory contains a summary report of the quality assessment and alignment steps. In the outputR directory, reports of the statistical analyses (DRIMSeq_dtu.html and edgeR_dge.html), as well as a list of SingleCellExperiment objects (in shiny_sce.rds) are saved. The latter can be imported into R and used for further downstream analysis. Using the template run_iSEE.R (available from https://github.com/csoneson/ARMOR/blob/chiron_realdataworkflow/E-MTAB-7029/run_iSEE.R) and shiny_sce.rds (available from https://doi.org/10.6084/m9.figshare.8040239.v1), an R/shiny web application can be initiated, with various panels to allow the user to interactively explore the data and results (Figure 5).

**Figure 6:**
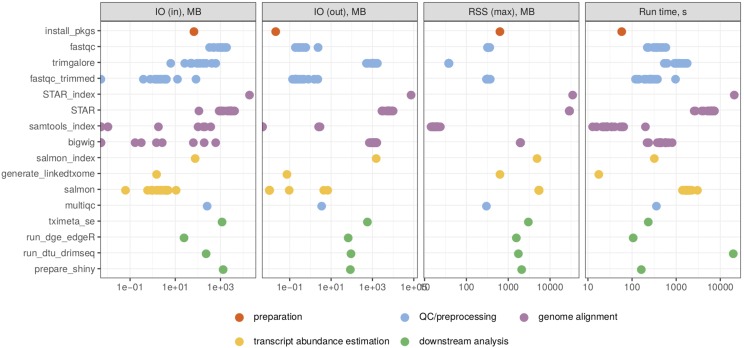
The required resources for the generation of each output file (grouped by the Snakemake rule) in the real data walk-through, as reported by the benchmarking directive of Snakemake. The four panels show the read and written bytes (in MB), the memory usage (in MB) and the run time (in seconds) of each rule. RSS = Resident Set Size. IO = Input/Output.

Figure 6 shows the run time and maximal memory usage for generating each output file. Note that the ncores parameter in the configuration file was kept at 1, and thus each rule was run using a single thread. The most memory-intensive parts of the workflow, due to the large size of the reference genome, were the generation of the STAR index and the alignment of reads to the genome. The most time consuming parts were the generation of the STAR index and the DTU analysis with DRIMSeq. However, both of these can be executed using multiple cores, by increasing the value of the ncores parameter.
